# Coordinated Transport of Nitrate, Potassium, and Sodium

**DOI:** 10.3389/fpls.2020.00247

**Published:** 2020-03-06

**Authors:** Natalia Raddatz, Laura Morales de los Ríos, Marika Lindahl, Francisco J. Quintero, José M. Pardo

**Affiliations:** Institute of Plant Biochemistry and Photosynthesis, Consejo Superior de Investigaciones Científicas and Universidad de Sevilla, Seville, Spain

**Keywords:** plant nutrition, salinity, potassium, nitrate, sodium, long-distance transport

## Abstract

Potassium (K^+^) and nitrogen (N) are essential nutrients, and their absorption and distribution within the plant must be coordinated for optimal growth and development. Potassium is involved in charge balance of inorganic and organic anions and macromolecules, control of membrane electrical potential, pH homeostasis and the regulation of cell osmotic pressure, whereas nitrogen is an essential component of amino acids, proteins, and nucleic acids. Nitrate (NO_3_^–^) is often the primary nitrogen source, but it also serves as a signaling molecule to the plant. Nitrate regulates root architecture, stimulates shoot growth, delays flowering, regulates abscisic acid-independent stomata opening, and relieves seed dormancy. Plants can sense K^+^/NO_3_^–^ levels in soils and adjust accordingly the uptake and root-to-shoot transport to balance the distribution of these ions between organs. On the other hand, in small amounts sodium (Na^+^) is categorized as a “beneficial element” for plants, mainly as a “cheap” osmolyte. However, at high concentrations in the soil, Na^+^ can inhibit various physiological processes impairing plant growth. Hence, plants have developed specific mechanisms to transport, sense, and respond to a variety of Na^+^ conditions. Sodium is taken up by many K^+^ transporters, and a large proportion of Na^+^ ions accumulated in shoots appear to be loaded into the xylem by systems that show nitrate dependence. Thus, an adequate supply of mineral nutrients is paramount to reduce the noxious effects of salts and to sustain crop productivity under salt stress. In this review, we will focus on recent research unraveling the mechanisms that coordinate the K^+^-NO_3_^–^; Na^+^-NO_3_^–^, and K^+^-Na^+^ transports, and the regulators controlling their uptake and allocation.

## Introduction

Plants take up essential nutrients and other minerals from the soil in various chemical forms. Some of them (K^+^ or NO_3_^–^) are essential for growth and taken in large quantities if available, while others (Na^+^ or NH_4_^+^) are potentially toxic at high concentrations. Contrary to nitrate and phosphate, K^+^ is not incorporated into organic matter, and hence it is the most abundant cation in tissues of well-fed plants, constituting between 2 to 10% of the dry weight of the plant (Leigh, 2001).

The physiological function of K^+^ ions include enzyme activation, osmotic regulation, turgor generation, cell expansion, pH homeostasis, regulation of electrical membrane potentials and electrical neutralization of the abundant negative charges within cells (Clarkson and Hanson, 1980; Pettigrew, 2008; Hawkesford et al., 2012; Zorb et al., 2014). Thus, large quantities of K^+^ are taken up from the soil solution by root epidermal and cortical cells, and then distributed throughout the plant. The concentration of K^+^ in the cytoplasm is kept rather constant, typically in the range of 75−100 mM when measured as K^+^ activity by ion-selective microelectrodes in several species (Maathuis and Sanders, 1993; Walker et al., 1996; Leidi et al., 2010; Planes et al., 2015). The vacuolar K^+^ pool is highly dynamic and serves as a repository that is replenished in times of abundance or wasted to preserve the homeostatic cytosolic concentration upon starvation (Martinoia et al., 2012; Ahmad and Maathuis, 2014). In barley roots, cytosolic K^+^ content began to decline only after the total tissue concentration dropped below 25 mM, while vacuolar concentrations ranged widely from 10 to 125 mM depending on the K^+^ status of the plant (Walker et al., 1996). Despite the homeostatic design to preserve optimal cytosolic K^+^ levels, both abiotic and biotic stresses result in the disturbance of intracellular K^+^ levels (Shabala and Pottosin, 2014). Relatively small changes in K^+^ concentration have profound effects on the electrical charge of the plasma membrane, which in turn initiates signaling events that trigger pertinent responses in K^+^ acquisition (Rubio et al., 2014), salinity (Leidi et al., 2010; Shabala, 2017), plant immunity (Brauer et al., 2016), and programmed cell death (Demidchik et al., 2010). Consequently, a signaling role has been proposed for the shifting levels of cytosolic K^+^ (Shabala, 2017).

Nitrogen is another macronutrient required by plants in the greatest amounts for optimal growth, and incorporated into numerous organic compounds (Mengel et al., 2001). For most plants, NO_3_^–^ and NH_4_^+^ are the prevalent nitrogen sources (Crawford, 1995; Gazzarrini et al., 1999). To be assimilated, NO_3_^–^ has to be taken up from the soil and converted into ammonium by nitrate and nitrite reductases, and then incorporated into amino acids via the glutamine-synthetase and glutamate synthase (GS-GOGAT) pathway. On the other hand, ammonium, as nitrogen source, is preferred over nitrate by most plants, but ammonium uptake through roots is tightly controlled because an elevated ammonium concentration in the cytosol becomes toxic to the plant (Gazzarrini et al., 1999; Straub et al., 2017). Potassium plays an essential role as counter-ion of NO_3_^–^, facilitating the uptake, translocation, and distribution of these ions between roots and shoots (Engels and Marschner, 1993; Zhang et al., 2010; Rodenas et al., 2017). Hence, the acquisition rates of K^+^ and NO_3_^–^ are often positively correlated (reviewed by Coskun et al., 2017). Under nutrient-sufficient conditions, K^+^:NO_3_^–^ co-translocation from the root-to-shoot is enhanced, while on the contrary, under nutrient-limited conditions the transport of both nutrients is restricted (Pettersson, 1984; Lin et al., 2008; Drechsler et al., 2015; Meng et al., 2016). The amount of supplied N and K must also be balanced to achieve maximum growth (Coskun et al., 2017). However, the mechanistic basis for the mutual influences exerted by these nutrients is poorly understood. By contrast, NH_4_^+^ is a strong inhibitor of the high-affinity K^+^ uptake by roots and translocation to shoots (Scherer et al., 1984; Wang et al., 1996; Spalding et al., 1999; Santa-Maria et al., 2000; ten Hoopen et al., 2010).

Sodium is the 7th most abundant element in the earth’s crust (2.4 vs. 2.1% of K^+^), present in all soils and surface and subterranean water bodies. However, unlike K^+^ and NO_3_^–^, it is not essential for either development or for the reproduction of plants with the exception of a subgroup of C4 plants that require traces of Na^+^ to drive the Na^+^-pyruvate co-transporter chloroplasts (Furumoto et al., 2011). In all other plants, this function is mediated by a H^+^-coupled pyruvate carrier. Under typical physiological conditions, plants maintain a high cytosolic K^+^:Na^+^ ratio with relatively low Na^+^ concentrations (20–30 mM) (Carden et al., 2003; Rodriguez-Navarro and Rubio, 2006; Kronzucker et al., 2013). However, as the ionic radii of Na^+^ and K^+^ in their hydrated forms are similar, under sodic conditions a failure in the discrimination among them often occurs, thus facilitating the Na^+^ influx through pathways that generally function for K^+^ uptake (Benito et al., 2014). The accumulation of toxic concentrations of Na^+^ in cells may have harmful effects, such as induction of cytosolic K^+^ efflux from both root and leaf cells and, subsequently an imbalance in cellular homeostasis, oxidative stress, interference with Ca^2+^ and K^+^ functions, disruption of protein synthesis, retarded growth and even plant death (Tester and Davenport, 2003; Munns and Tester, 2008; Craig Plett and Moller, 2010; Cabot et al., 2014).

Considering the extent and physiological importance of these interactions between NO_3_^–^, K^+^, and Na^+^, in this review we describe the operation and diversity of the main mechanisms that coordinate the K^+^-NO_3_^–^, Na^+^-NO_3_^–^, and K^+^-Na^+^ transports, and their regulators that control their uptake and movements. Most of the proteins and processes described herein belong to *Arabidopsis thaliana* and rice because of the wealth of information available in these model species.

## Potassium–Nitrate Interactions

In most plant species, the uptake rates of K^+^ and NO_3_^–^ from the soil are positively correlated and to enhance one another. This effect can be explained by the improved charge balance during nutrient uptake and long-distance transport and by the K^+^-induced activation of the enzymes involved in nitrate assimilation. Consequently, plants grown in the presence of NO_3_^–^ take up and accumulate more K^+^ than when grown with NH_4_^+^. However, little is known about the direct influences produced by one ion on the transport of the other (Coskun et al., 2017).

To cope with variable nitrate concentrations in soil, tissues and within cells, plants have developed both a High-Affinity Transport System (HATS; K_*m*_ in the μM range) and a Low-Affinity Transport System (LATS; K_*m*_ of mM) for the acquisition and distribution of nitrate. When the external nitrate concentration is high (e.g., >1 mM), LATS is preferentially used; otherwise, the inducible HATS are activated and take over nitrate transport (Glass et al., 1992; Crawford and Glass, 1998). Two protein families, NRT1/NPF and NRT2, have been identified as responsible for LATS and HATS, respectively. Exceptions are NRT1.1, which has a dual high- and low-affinity for nitrate, depending on the phosphorylation state, and NRT2.7 which despite belonging to NRT2 family, shows low nitrate affinity (Glass et al., 1992; Orsel et al., 2002; Chopin et al., 2007; Tsay et al., 2007). Some endosomal channel-like exchangers of the CLC family, and the slow anion channels SLAC1/SLAH also transport nitrate. Collectively, these four families of anion transporters amount to 70 genes in *A. thaliana*, albeit just a few of them have been confirmed to transport nitrate (Fan et al., 2017).

**FIGURE 1:**
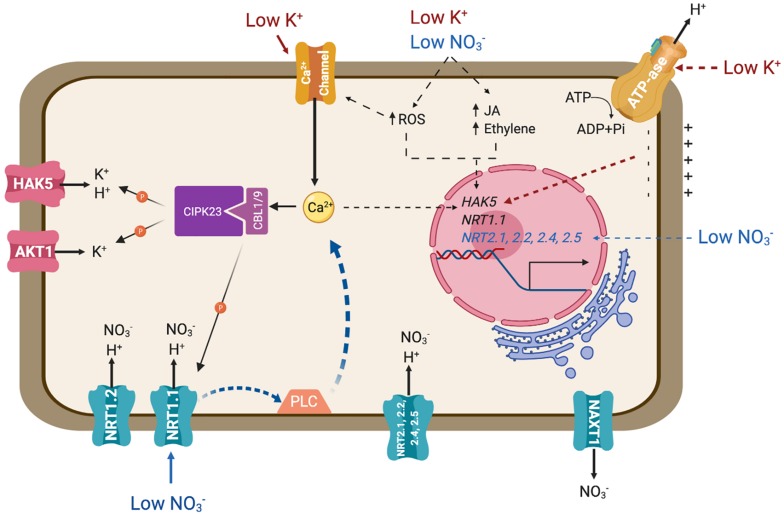
Transporters involved in the main pathways for the uptake of nitrate and potassium. The diagram represents an idealized root cell disregarding developmental differentiation. Major signaling pathways regulating the expression and biochemical activity of these transporters are also represented. Solid lines represent signaling events and connections that have been confirmed experimentally. Dotted lines signify known or suspected connections for which the molecular events involved remain to be defined. PLC represents an unidentified phospholipase C. Further details are given in the main text.

The NRT1/NPF family shares significant sequence identity to mammalian and bacterial PTR peptide transporters. The NRT1/NPF family belongs to the Major Facilitator Superfamily (MFS) of secondary active transporters that use the proton electrochemical gradient to drive substrate uptake into cells. Although several members of the NRT1/NPF have been shown to mediate nitrate transport, other members of this large family may not be competent for this process but instead mobilize diverse substrates ranging from dipeptides to hormones, including ABA and auxin (Leran et al., 2015a; Fan et al., 2017). NRT1.1/CHL1 is the most studied nitrate transporter and represents a major pathway for nitrate uptake (Tsay et al., 1993, 2007; Figure 1). Notably, the Arabidopsis NRT1.1 is a dual-affinity nitrate transporter that also serves as a sensor for its substrate (Wang et al., 1998). In conditions of high nitrate availability (>1mM) NRT1.1 behaves as a low-affinity transporter (K_*m*_ ∼4 mM). However, when nitrate levels fall below 1 mM, NRT1.1 is phosphorylated by the CIPK23 protein kinase, switching into a high-affinity mode (K_*m*_ ∼40 μM) (Wang et al., 1998; Liu et al., 1999; Ho et al., 2009). NRT1.2, expressed in root epidermis and cortex also contributes in low-affinity nitrate uptake, together with other LATS yet to be identified (Figure 1; Huang et al., 1999; Nacry et al., 2013). On top of the nitrate transport activity of NRT1.1, this sensor protein governs essential physiological, developmental and molecular features of the plant response to nitrate availability by means of its capacity for auxin transport. Under low nitrate conditions, NRT1.1 functions to take up and remove auxin from the lateral root primordia, thus repressing the development of lateral roots. Nitrate inhibits NRT1.1-dependent auxin uptake, which in turn stimulates lateral root development (Krouk et al., 2010). Mutations in residues P492 and T101, the later being phosphorylated by CIPK23, decrease auxin transport of NRT1.1 and impair the regulation of lateral root development (Bouguyon et al., 2015, 2016).

The *NRT2* family consists of seven members in the *Arabidopsis* genome. NRT2.1, NRT2.2, NRT2.4 and NRT2.5 are involved in inducible high-affinity nitrate uptake (Figure 1; Cerezo et al., 2001; Li et al., 2007; Kiba et al., 2012; Kiba and Krapp, 2016). These four NRT2 transporters are responsible of approximately 95% of high-affinity nitrate influx activity under nitrate-limited conditions, as evidenced by the phenotype of the quadruple mutant *nrt2.1/nrt2.2/nrt2.4/nrt2.5* (Lezhneva et al., 2014). Although NRT1 and NRT2 proteins are functionally and phylogenetically distinct, both are believed to couple nitrate and proton translocation to sustain nitrate transport regardless of the thermodynamical constraints imposed by the nitrate gradient across biological membranes (Paulsen and Skurray, 1994; Orsel et al., 2002). Passive efflux, i.e., downward the electrochemical gradient of nitrate, is facilitated by channels, including SLAH3 in guard cells (Geiger et al., 2011; Zheng et al., 2015). Efflux in the cortex of mature roots is achieved by the electroneutral NO_3_^–^/H^+^ symporter NAXT1/NPF2.7 (Figure 1; Segonzac et al., 2007). The biological role of this nitrate leak leading to a decrease in root NO_3_^–^ content is unclear because interference with *NAXT1* gene expression did not reveal a role in plant N nutrition in standard culture conditions (Segonzac et al., 2007). A NAXT-like protein, NPF2.3, contributes to nitrate efflux in the root pericycle and loading into the xylem sap (Taochy et al., 2015).

**FIGURE 2:**
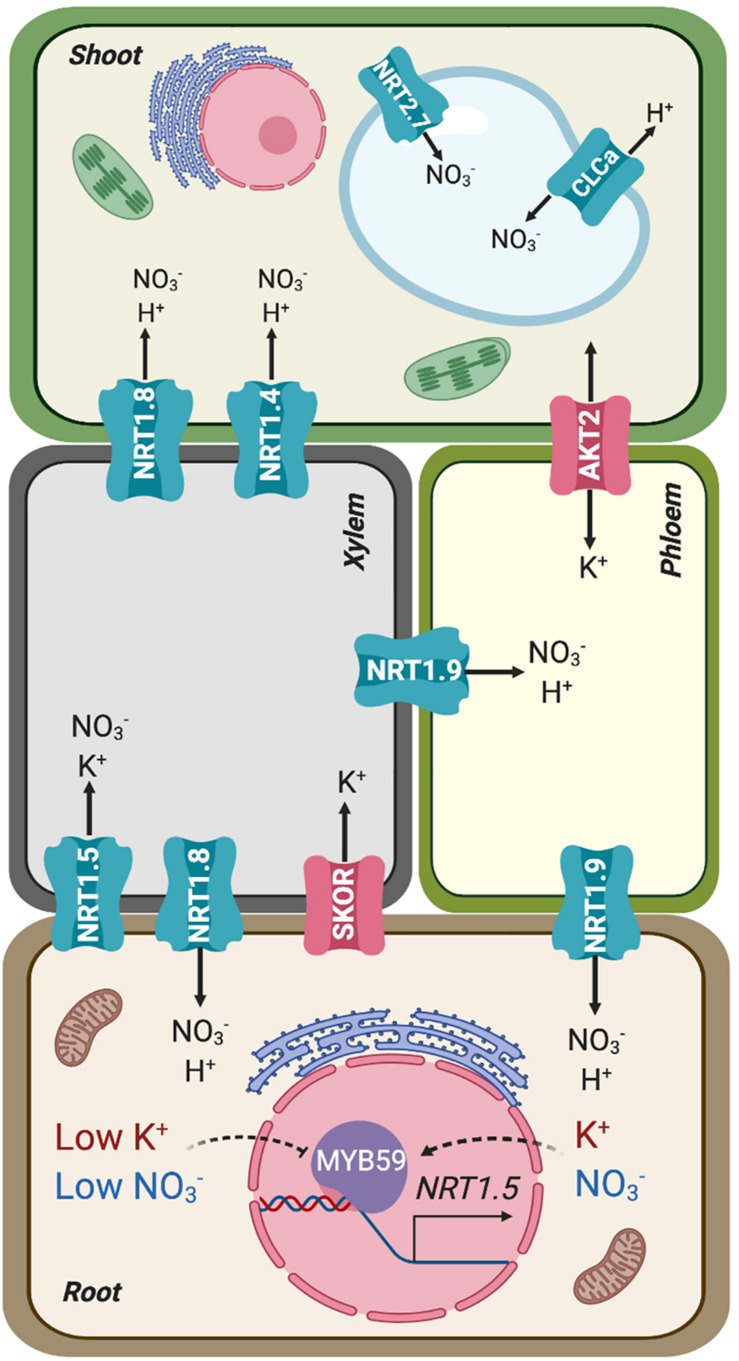
Simplified representation of the source (root) and sink (shoot) organs in terms of mineral uptake and distribution, connected by xylem and phloem. Transporters involved in the root-shoot partition of nitrate and potassium are represented in their preferential placement based on gene expression and protein activity. AKT2 is represented as bidirectional owing to its facultative inward-rectification.

Long-distance transport of nitrate involves xylem loading and unloading, two successive steps that determine net distribution and assimilation efficiency (Figure 2; Krapp, 2015). After entering the root cytoplasm, nitrate can be loaded into xylem vessels by NRT1.5, expressed in root pericycle cells, and subsequently retrieved from the xylem sap in plant roots and aerial tissues by NRT1.8, expressed predominantly in xylem parenchyma cells (Lin et al., 2008; Li et al., 2010). Under stress conditions (salinity, drought, and cadmium treatment), *NRT1.5* expression in roots decreases and nitrate loading into xylem vessels is reduced. By contrast, *NRT1.8* expression in roots increases, enhancing nitrate unloading back into roots. This coordinated regulation is mediated by ethylene and jasmonic pathways (Li et al., 2010; Zhang et al., 2014). Once nitrate has reached the aerial tissues, the low-affinity nitrate transporter NRT1.4, preferentially expressed in leaf petioles, gates nitrate distribution within leaves (Figure 2). The activity of NRT1.4 contributes to cell expansion. Under high nitrate conditions, NRT1.9 mediates nitrate transport back to roots via phloem (Figure 2). This mechanism prevents excess amounts of nitrate being accumulated in shoots (Wang and Tsay, 2011). Moreover, nitrate can be remobilized from older leaves to feed young leaves via NRT1.7, expressed in the phloem of minor veins (Fan et al., 2009). At destination, nitrate is either stored inside vacuoles using K^+^ as counterion, where both ions contribute to osmotic adjustment (Barragan et al., 2012; Martinoia et al., 2012), or reduced to nitrite and then partitioned into plastids to be assimilated to organic nitrogen (Wang et al., 2012, 2018). The low-affinity nitrate transporter NRT2.7 and the channel-like NO_3_^–^/H^+^ exchanger CLCa were identified as responsible for nitrate translocation into vacuoles (De Angeli et al., 2006; Chopin et al., 2007; Figure 2). The transporters responsible for exporting nitrate out of vacuoles remain to be identified.

Potassium uptake by roots often exhibits complex biphasic kinetics in response to increasing external concentrations. At least two transport systems are involved in potassium uptake, corresponding to high- and low- affinity transports systems, which work at low (<1 mM) and high (>1 mM) external K^+^ concentrations, respectively (Nieves-Cordones et al., 2014; Ragel et al., 2019). At high concentrations outside, K^+^ crosses the plasma membrane mostly through selective channels, e.g., the *Shaker*-like channel AKT1 (Lagarde et al., 1996; Nieves-Cordones et al., 2016; Figure 1). At low K^+^ concentrations, proton-coupled transport systems, such as HAK5 of Arabidopsis and HAK1 of rice, are needed in order to pull potassium inside cells against its electrochemical gradient (Gierth et al., 2005; Nieves-Cordones et al., 2016; Santa-Maria et al., 2018). The cryo-EM structure of KimA, a KUP-like protein from *Bacillus subtilis*, has been resolved recently (Tascon et al., 2020). The structure shows a homodimer alternating between occluded and opened arrangements formed by tilted protomers that likely rock with a “breading” motion during K^+^/H^+^ symport.

Plants respond to K^+^ availability by different means. Hyperpolarization of the root cell membrane is considered to be the earliest signaling event elicited by K^+^ deficiency (Nieves-Cordones et al., 2008; Figure 1). Also associated to K^+^ starvation are increases in cytosolic calcium (Ca^2+^), the alteration of different hormone levels (such as ethylene and jasmonate), and production of reactive oxygen species (ROS) (Armengaud et al., 2004; Shin and Schachtman, 2004; Jung et al., 2009; Behera et al., 2017; Figure 1). Together, these stimuli lead to transcriptional and post-translational regulation of K^+^ uptake systems. At the transcriptional level, the *HAK5* transporter is activated by K^+^ starvation, specifically responding to hyperpolarization of the plasma membrane (Nieves-Cordones et al., 2008), and quickly repressed after K^+^ supply (Ahn et al., 2004; Gierth et al., 2005; Aleman et al., 2009). At the post-transcriptional level, Ca^2+^ signaling under K^+^ deprivation is registered by the CBL1/CBL9 Ca^2+^ sensors, that activate and recruit the kinase CIPK23 to the plasma membrane to achieve the phosphorylation and activation of both AKT1 and HAK5 transporters (Li et al., 2006; Xu et al., 2006; Ragel et al., 2015; Figure 1). HAK5 activation produced an increase in the affinity and the V_*max*_ of K^+^ transport, ensuring the entry of K^+^ inside the cell at concentrations lower than 0.1 mM (Nieves-Cordones et al., 2014; Ragel et al., 2015), whereas phosphorylation of AKT1 results in channel activation that maximizes K^+^ influx (Geiger et al., 2009).

The CIPK23/CBL1-9 module not only phosphorylates and activates K^+^ uptake systems AKT1 and HAK5, but also mediates high- and low-affinity transition of the nitrate transporter and sensor (transceptor) NRT1.1 (Ho et al., 2009; Leran et al., 2015b; Figure 1). The crystal structure of NRT1.1 reveals a biologically relevant dimer, whose dynamic coupling and decoupling of monomers is controlled by the phosphorylation of a single residue, Thr101, by CIPK23 (Ho et al., 2009; Parker and Newstead, 2014; Sun et al., 2014). This residue is strictly conserved among plant NRT1.1 orthologs. Non-phosphorylated NRT1.1 is a low-affinity nitrate transporter working as a dimer. According to the common view, at low external nitrate concentration, a Ca^2+^ signaling cascade leads to the phosphorylation of NRT1.1 by CIPK23/CBL1-9 and dimer dissociation. Phosphorylated NRT1.1 monomers show a higher nitrate affinity than the dimers (Tsay et al., 2011; Li et al., 2017). These findings bring about two questions. One is how the CIPK23/CBL1-9 complex is capable of resolving different nutrient-related stimuli and then targets the pertinent K^+^ or nitrate transporter. One reason could be the sequence of events leading to transporter phosphorylation/activation. Rashid et al. (2019) proposed that nitrate binding by only one NRT1.1 monomer triggers dimer dissociation and exposes the Thr101 residue to enable phosphorylation by CIPK23. This phosphorylation stabilizes the monomeric state of NRT1.1. However, at higher nitrate concentrations, substrate binding by both monomers promotes NRT1.1 dimerization, which attenuates CIPK23 activity and thereby maintains the low-affinity mode of nitrate signaling and transport (Rashid et al., 2019). Moreover, a functional NRT1.1 is necessary to trigger nitrate-induced Ca^2+^ waves through the action of an unknown phospholipase C (Riveras et al., 2015; Figure 1). Whether this kinetic model also applies to HAK5 and AKT1, which likely are dimers and tetramers themselves (Daram et al., 1997; Daras et al., 2015; Tascon et al., 2020), is unknown. In K^+^ transport, it is assumed that K^+^ starvation elicits a Ca^2+^ signal perceived by CBL1 and CBL9 (Behera et al., 2017), which then recruit CIPK23 to the plasma membrane to phosphorylate and activate AKT1 and HAK5 transporters. In other words, Ca^2+^-induced phosphorylation of K^+^ transport protein leads to conformational and kinetics changes, whereas for NRT1.1 the low availability of the substrate is what induces the phase transition from dimers to monomers. This, in turn, facilitates phosphorylation to stabilize the new conformation, which also elicits a Ca^2+^ signal that reinforces the output by stimulating CBL1/9-dependent CIPK23 activity (Rashid et al., 2019). How those alternative models apply to ammonium, magnesium and iron transporters, and channels SLAC1 and SLAH3, all of which are also regulated by CIPK23, is unclear (Maierhofer et al., 2014; Tang et al., 2015; Straub et al., 2017; Dubeaux et al., 2018).

The second, broader question is why nitrate and potassium transporters need to be regulated by the same kinase in the first place. The answer to this question likely relates to the tight linkage between K^+^ and nitrate uptake and distribution. The expression of *NRT1.1* in roots is enhanced by low K^+^-treatment, and the transporter is required for plants to resist K^+^ deficiency under sufficient NO_3_^–^ in concert with K^ +^ uptake channels (Fang et al., 2019). In these conditions, the *nrt1.1* knockout mutant exhibited severe leaf senescence, shorter roots and less biomass than Col-0 plants, while the quadruple mutant *nrt2.1*, *nrt2.2*, *nrt2.4*, and *nrt2.5* lacking several high-affinity nitrate transporters showed a phenotype similar to wild-type plants. In addition, the rates of root Rb^+^ uptake (the closest analog of K^+^) in *nrt1.1* mutant were considerably less than those in Col-0 plants in low-Rb^+^ medium. How low-K^+^ stress up-regulates NRT1.1 activity is not yet known, but it likely involves activation of the CIPK23/CBL1-CBL9 module.

Potassium and nitrate are also linked with each other in their translocation to shoots (Figure 2). In general, under nutrient sufficient conditions, the root-to-shoot transport of K^+^ and NO_3_^–^ is enhanced (Coskun et al., 2017). Conversely, co-translocation is restricted in plants under limited availability of either nutrient. However, under low-NO_3_^–^ and K^+^-sufficient conditions, NO_3_^–^ can be partially substituted by other anions such a chloride, indicating that charge balance is a key factor behind the observed linkage. The cooperative translocation of K^+^ and NO_3_^–^ via the vasculature has been interpreted as an internal ion cycling by which NO_3_^–^ is transported from root to shoot using K^+^ as counterion in the xylem sap. In the shoot NO_3_^–^ is assimilated into amino acids and organic acids. Malate is then transported to roots via the phloem, again accompanied by K^+^ as counterion (Zioni et al., 1971; Kirkby and Knight, 1977; Touraine et al., 1988; Engels and Kirkby, 2001). Potassium circulating in the phloem is also involved in supporting sucrose transport from source to sink tissues. The K^+^ channel AKT2, a facultative inward-rectifier controlled by phosphorylation (Figure 2), energizes sucrose loading into the phloem of Arabidopsis (Michard et al., 2005; Gajdanowicz et al., 2011).

Molecular mechanisms that directly coordinate the long-distance transport of NO_3_^–^ and K^+^ are beginning to emerge. To transport ions to the shoot, they must be loaded into the xylem vessels of the root vascular stele (Ahmad and Maathuis, 2014). Until recently, only the K^+^ channel SKOR had been implicated in root-to-shoot K^+^ translocation in *Arabidopsis* (Gaymard et al., 1998; Figure 2). SKOR belongs to the voltage-dependent *Shaker*-like superfamily of K^+^ channels. SKOR is expressed in the root pericycle and xylem parenchyma of *Arabidopsis*, and mediates K^+^ secretion into xylem vessels. At high external K^+^ concentration in the vasculature, the channel stabilizes in a closed state. However, with a low external K^+^ concentration and when the plasma membrane of xylem parenchyma cells becomes depolarized, SKOR opens and mediates the release of cellular K^+^ to the stele apoplast and xylem vessels. Since the cell interior is electrically negative relative to the exterior, the uptake of the nitrate anion driven by the co-transport of two protons would initially depolarize the plasma membrane. Likewise, the efflux of anions, such as NO_3_^–^ or Cl^–^, out of parenchyma cells in the stele could lead to membrane depolarization that in turn would elicit K^+^ release via SKOR, thereby explaining the observed linkage between anionic (NO_3_^–^, SO_4_^–^, Cl^–^) and cationic (K^+^, Ca^2+^, Mg^2+^) nutrients (Leigh, 2001; Drechsler et al., 2015). The *skor* mutation strongly reduced the K^+^ content in the shoot and xylem sap with little effect on the root K^+^ content (Gaymard et al., 1998), but surprisingly *skor* null mutants do not exhibit a particular K^+^-deficient phenotype, which suggests that other proteins may also participate in this process. Notably, one nitrate transporter, NRT1.5, has been shown to affect root-to-shoot K^+^ translocation under low NO_3_^–^ availability (Drechsler et al., 2015) and to be involved in K^+^ and NO_3_^–^ transport by xylem under K^+^ limited conditions (Li et al., 2017; Figure 2).

As said before, nitrate can be assimilated into ammonium and then to amino acids. A significant proportion of nitrate assimilation takes place in shoot because the reducing power required for the assimilation processes comes from photosynthesis (Searles and Bloom, 2003; Krapp, 2015). NRT1.5 of Arabidopsis was first identified as a *bona fide* nitrate transporter in *Xenopus* oocytes and shown to facilitate nitrate loading into xylem vessels (Lin et al., 2008). However, NRT1.5 has later been shown to operate as a proton-coupled H^+^/K^+^ antiporter (Li et al., 2017). Thus, at external acidic pH (as in the xylem sap) NRT1.5 promotes K^+^ release out of cells into the xylem. In *nrt1.5* mutants, the amount of K^+^ transported to the shoot is reduced in K^+^-sufficient and K^+^- deficient conditions, while the mutant *nrt1.5* accumulates higher K^+^ and NO_3_^–^ in the root under K^+^-deficient conditions (Lin et al., 2008; Li et al., 2017). According to the crystal structure of NRT1.1, which is an electrogenic NO_3_^–^/H^+^ symporter, the “ExxER” motif (containing three conserved residues) on transmembrane TM1 together with the conserved residue Lys-164 on TM4 is responsible for proton coupling (Parker and Newstead, 2014). These four charged residues are highly conserved in most Arabidopsis NRT1 members. By contrast, NRT1.5 only has non-charged residues in these four sites, suggesting that this transporter must have an alternative proton-coupling mechanism compared with other NRT1 members. One possibility that deserves further exploration is whether differences in amino acids related to proton-binding in NRT1.1 allow NRT1.5 to couple the co-transport K^+^ and nitrate as substrates. Despite the controversial mechanism of transport by NRT1.5, the consensus is that NRT1.5 affects the homeostatic balance between K^+^ and nitrate in the xylem stream (Lin et al., 2008; Li et al., 2017). Inactivation of *NRT1.5* also promoted the expression of genes responsive to phosphate deficiency and increased the concentration of phosphate in tissues compared to wild-type plants under phosphate starvation. However, this appeared to be an indirect effect of ethylene production in the mutant since inhibition of ethylene synthesis canceled differences between *nrt1.5* and wild-type plants with regard to the phosphate response (Cui et al., 2019).

Coordinated regulation between K^+^ and NO_3_^–^ in translocation to xylem also exists at the transcriptional level. The expression of genes *SKOR* and *NRT1.5* was up-regulated by nitrate supply. During low-K^+^ stress, the *NRT1.5* transcript is down-regulated, presumably to adjust root-to-shoot K^+^/NO_3_^–^ transport to K^+^ levels (Wang et al., 2004; Lin et al., 2008; Li et al., 2017). Similar results have been reported in rice regarding the nitrate transporter OsNPF2 expressed in the root epidermis, xylem parenchyma, and phloem companion cells (Xia et al., 2015). Knockout of *OsNPF2.4* decreased K^+^ concentration in xylem sap. Conversely, K^+^ deprivation resulted in the up-regulation of the nitrate transporters *NRT1*.*2* and *NRT2*.*1* in tomato roots (Wang et al., 2001) and of NRT1.1 in Arabidopsis (Armengaud et al., 2004).

Recently, the Arabidopsis transcription factor *MYB59* has been shown to positively regulate *NRT1.5* expression and to balance K^+^/NO_3_^–^ transport (Du et al., 2019). Under K^+^/NO_3_^–^ sufficient conditions, MYB59 binds to the *NRT1.5* promoter and facilitates NRT1.5-mediated root to shoot K^+^/NO_3_^–^ transport (Figure 2). When plants are subjected to K^+^/NO_3_^–^ deficient conditions, *MYB59* is down-regulated, which subsequently impairs the accumulation of the *NRT1.5* transcript. These data further support a co-regulation at the level of xylem transport that maintains the balance between NO_3_^–^ and K^+^.

**FIGURE 3:**
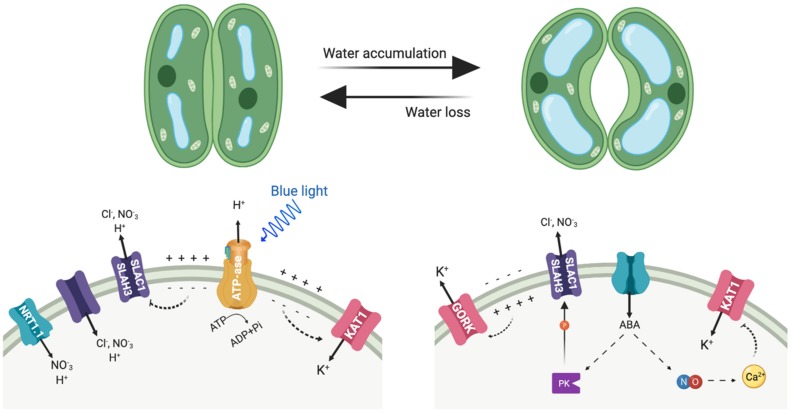
Depicted are the main transporters mediating anion and cation fluxes at the plasma membrane of guard cells and involved in stomatal movements. Left side, stomata opening; right side, stomata closure. PK represents protein kinases, including OST1 and CPK3.

Guard cells represent a paradigmatic example of how NO_3_^–^ and K^+^ transport are functionally linked at the cellular level (Figure 3). Stomata consist of pairs of guard cells that dynamically and reversibly change their turgor and volume to adjust the size of the stomatal pore. This is accomplished by the massive uptake and release of K^+^ and nitrate ions among other solutes, and by the biosynthesis of organic compounds (Eisenach and De Angeli, 2017; Hedrich and Shabala, 2018). To open the stomata, firstly the activation of H^+^-ATPase AHA1 hyperpolarizes the plasma membrane, negative inside, which then triggers the influx of K^+^ into the cytoplasm following the electrochemical gradient (Figure 3). The influx of K^+^ partly depolarizes the membrane, which in turn favors that charge-balancing anions accumulate in the guard cell (Yamauchi et al., 2016; Jezek and Blatt, 2017). The inward-directed H^+^ gradient allows the symport of sugars, and of organic (malate) and inorganic anions (Cl^–^, NO_3_^–^) in co-transport with H^+^ (Eisenach and De Angeli, 2017; Jezek and Blatt, 2017). This increased uptake of osmolytes triggers water influx, inflates the guard cells and the stomatal pore opens. NRT1.1, which is expressed not only in roots but also in the guard cells, contributes to promoting stomatal opening (Guo et al., 2003). Conversely, stomatal closure activates the anion channels SLAC1 and SLAH3, which differ in their Cl^–^/NO_3_^–^ permeability and are activated by a distinct set of protein kinases that are stimulated by ABA (Vahisalu et al., 2008; Geiger et al., 2009, 2010, 2011; Lee et al., 2009; Figure 3). As result of anion exit through these channels, the plasma membrane depolarizes. This voltage drop at the plasma membrane activates the potassium outward-rectifying channel GORK, what finally leads to K^+^ efflux for decreasing turgor and stomatal closure.

Abscisic acid (ABA) promotes stomatal closure at least in part by driving the increase in nitric oxide, which in turn leads cytosolic calcium elevation (Chen et al., 2016). The effects in elevating cytosolic calcium results in the suppression of currents at the plasma membrane through the K^+^ inward channel to prevent K^+^ influx and activation of K^+^ outward and anion channels for ion efflux and stomatal closure. The main source of nitric oxide is the reduction of nitrite to nitric oxide, catalyzed by two nitrate reductases encoded by *NIA1* and *NIA2* genes (Wilson et al., 2008). Accordingly, stomatal opening is significantly affected in the double mutant *nia1 nia2* in normal growth conditions throughout the day. Beside this, *nia1 nia2* was unable to fully open its stomata even under high external K^+^, suggesting the mutations may affect guard cell K^+^ transport, as K^+^ is the main solute for stomatal opening (Chen et al., 2016).

## Nitrate–Sodium Interactions

Substantial interactions between nitrate and sodium transport could be expected in marine plants and algae thriving in a medium with high salinity and moderately alkaline pH (pH 7.5–8.4). Nitrate is present at low (1–10 μM) concentrations in seawater and must be captured against a steep electrochemical gradient across the plasma membrane, which theoretically could be coupled to the co-transport of H^+^ or the abundant Na^+^ ions (Rubio et al., 2005). However, only few reports have described Na^+^-linked nutrient uptake in marine plants. One example is the seagrass *Zostera marina*, which evolved from a terrestrial angiosperm that returned to the sea. Like extant land species, the ancestor of *Z. marina* presumably transported NO_3_^–^ from the soil using H^+^-coupled transport systems. Therefore, an interesting question is how Na^+^-coupled NO_3_^–^ transport evolved in *Z. marina* (Garcia-Sanchez et al., 2000). Identifying the transporter(s) involved in Na^+^/NO_3_^–^ co-transport could potentially yield important structural information regarding the ion selectivity of nitrate transporters.

Not surprisingly, there are very few reports about Na^+^-coupled transport systems in terrestrial plants. Na^+^-dependent nitrate transport has been described in the halophytes *Suaeda physophora* and *Salicornia europea* (Junfeng et al., 2010; Nie et al., 2015). In *Beta vulgaris*, Na^+^ enhances both nitrate uptake and translocation to shoots (Kaburagi et al., 2014, 2015). On the other hand, a large proportion of Na^+^ ions accumulated in Arabidopsis shoots were loaded into the xylem by transport systems that appeared to couple the movement of Na^+^ to that of nitrate (Alvarez-Aragon and Rodriguez-Navarro, 2017). The nitrate-dependent loading of Na^+^ into the xylem was additive to that of SOS1, a Na/H exchanger mediating Na^+^ efflux at the xylem parenchyma cells (Shi et al., 2002; El Mahi et al., 2019). Nitrate-dependent Na^+^ transport was partially interrupted in the *nrt1.1* mutant but not in *nrt1.2*, implying that unidentified nitrate transporters under the regulation of the NRT1.1 transceptor were involved in this process (Alvarez-Aragon and Rodriguez-Navarro, 2017). Notably, this linked Na^+^/NO_3_^–^ transport served the purpose of osmotic adjustment since it prevented the wilting of plants challenged with a hyperosmotic medium. The combined use of nitrate and chloride as permeable anions showed a predominant role of nitrate to stimulate Na^+^ accumulation, suggesting that nitrate fulfilled a specific function that chloride did not achieve. Thus, it appears that under high salinity Na^+^ may partly substitute for K^+^ in the extensive K^+^-NO_3_^–^ interactions described above, particularly in those connected to charge balance and the re-distribution of K^+^ as a cellular osmoticum.

Under stress conditions, a significant amount of nitrate assimilation into organic matter is shifted from shoots to roots (Krapp, 2015). As explained above, the coordinate action of NRT1.5/NPF7.3 and NRT1.8/NPF7.2 determines the root/shoot partition of nitrate in Arabidopsis. Upon salinity or heavy metal stress, expression of *NRT1.*5 in roots decreases to limit the nitrate load of xylem vessels, while that of *NRT1.*8 is induced to favor nitrate unloading back into the root symplasm (Li et al., 2010; Chen et al., 2012; Zhang et al., 2014). Cadmium and sodium stresses initiated ethylene (ET) and jasmonic acid (JA) signaling pathways, which promoted the binding of the ET-responsive transcription factors ERF59, *ERF1B*, and *ERF104* to the *NRT1.8* promoter, and of EIN3 to the *NRT1.5* promoter (Zhang et al., 2014). Moreover, EIN3 further induced the expression of ERF59, *ERF1B*, and *ERF104*, thereby acting as an integrator of ET and JA signaling.

The nitrate efflux protein NPF2.3 is preferentially expressed in the root pericycle, where it contributes to nitrate loading in the xylem together with NRT1.5/NPF7.3 (Taochy et al., 2015). *NPF2.3* gene disruption resulted in salt sensitivity and reduced nitrate translocation to shoots, but only under salt stress even though *NPF2.3* was expressed at similar levels to control conditions. The prevalence of NPF2.3 under salt stress may result from transcriptional repression of *NRT1.5/*NPF7.3. Presumably, the salinity-induced repression of *NRT1.5/NPF7.3* likely prevents detrimental Na^+^ accumulation in shoots since disruption of this gene led to decreased Na^+^ content in shoots and enhanced tolerance to salinity (Chen et al., 2012). Hence, it is unlikely that NRT1.5/NPF7.3 is involved in the nitrate-dependent Na^+^ transport reported by Alvarez-Aragon and Rodriguez-Navarro (2017) because these authors evidenced the beneficial effect of Na^+^ distribution along the plant axis to improve osmotic adjustment. On the other hand, translocation of nitrate to shoots by NPF2.3 proceeded without inducing any significant increase in shoot Na^+^ content, and growth impairment probably resulted from defective nitrate assimilation (Taochy et al., 2015). It remains to be resolved whether the patterns of Na^+^ distribution in *nrt1.5* and *npf2.3* mutants result from altered xylematic transport K^+^, an antagonist of Na^+^, as we discuss in the next Section, and whose transport is intimately connected to that of nitrate.

## Sodium–Potassium Interactions

Sodium and potassium interact at two main levels: the interference of Na^+^ with K^+^ nutrition, and the substitution of Na^+^ for K^+^ as highly dynamic and mobile cellular osmolyte in conditions of K^+^ shortage (Haro et al., 2010; Kronzucker and Britto, 2011; Kronzucker et al., 2013; Alvarez-Aragon et al., 2016). Soil salinity is often associated with elevated levels of Na^+^ (Munns and Tester, 2008). Although it is not clear what cytosolic levels of Na^+^ are harmful to the plant cell (Kronzucker and Britto, 2011; Alvarez-Aragon et al., 2016), this cation is usually excluded from the cytosol. Due to their physicochemical similarity, Na^+^ and K^+^ can compete for binding to amino acids of protein surfaces, pockets of allosteric regulation or selectivity filters of ion channels (Benito et al., 2014). As a result, high Na^+^ concentrations in plants trigger K^+^-deficiency symptoms and disrupt many physiological processes mediated by K^+^ such as protein synthesis and enzymatic reactions. Moreover, membrane depolarization caused by the entry of Na^+^ into the cell results in compromised K^+^ uptake through inward-rectifying K^+^ channels, making it thermodynamically unfavorable, together with the increased K^+^ efflux through outward-rectifying channels (Shabala et al., 2006; Coskun et al., 2013). Contrary to the debate regarding whether NRT proteins transport NO_3_^–^, K^+^ or both, it is clear that Na^+^ competes with K^+^ in plant uptake specifically through High-Affinity K^+^/K^+^ UPtake/K^+^ Transporter (HAK/KUP/KT), High-Affinity Potassium Transporters (HKTs) and Non-Selective Cation Channels (NSCCs) (Kronzucker and Britto, 2011).

**FIGURE 4:**
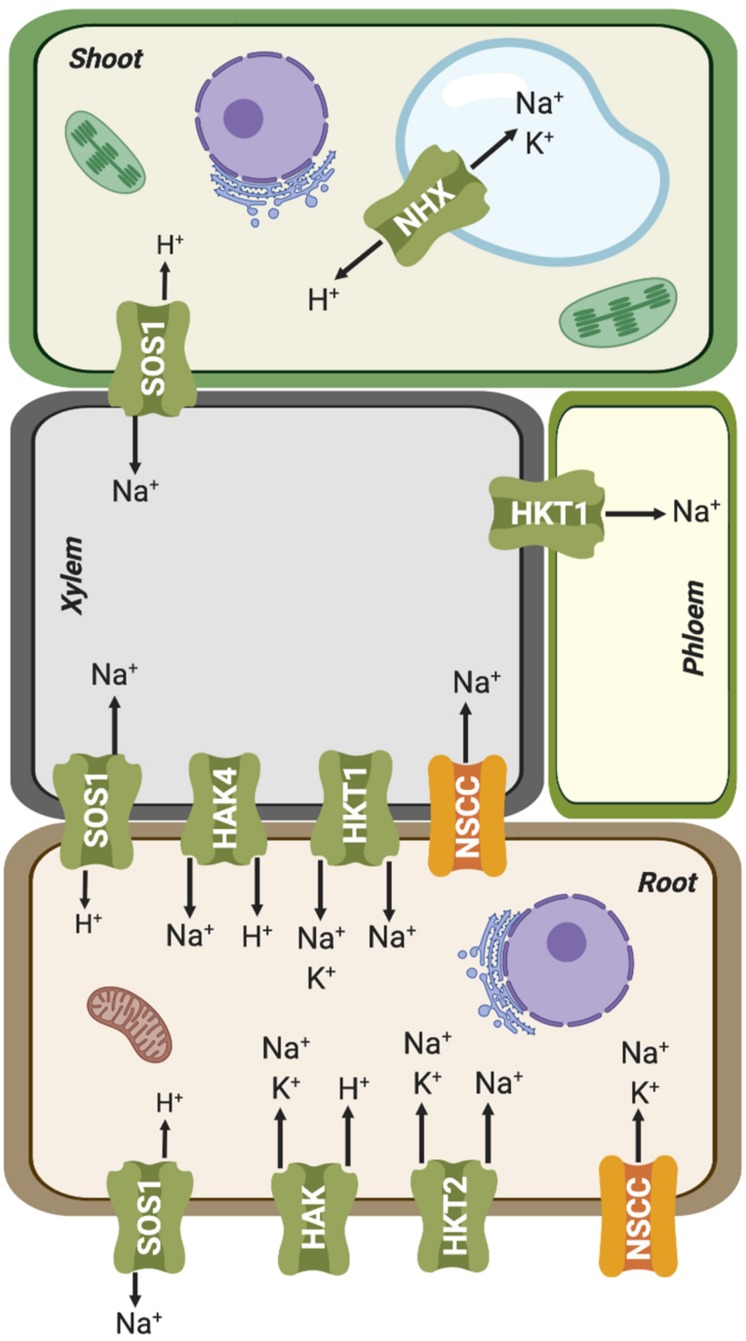
Simplified representation of plant organs and vascular tissues, and the Na^+^ and K^+^ fluxes mediated by ion transporters involved in root uptake and root-shoot partition. Further details are given in the main text.

HAK transporters are essential for K^+^ absorption related to mineral nutrition, root hair formation and adaptation to abiotic stresses (Osakabe et al., 2013; Nieves-Cordones et al., 2014; Very et al., 2014). However, they could also have an important role in enabling Na^+^ uptake. Indeed, different members of this family have been shown to mediate high-affinity Na^+^ uptake. PpHAK13 from the moss *Physcomitrella patens* transports Na^+^ but not K^+^. This transporter appears to be a major pathway for Na^+^ entry at low external concentration in *P. patens* because high-affinity Na^+^ uptake was abolished in the *hak13* knockout (Benito et al., 2012). PhaHAK2 from reed plants (*Phragmites australis*) is permeable to Na^+^, and its gene induced by low-K^+^ conditions but repressed under salt stress, thereby limiting toxic Na^+^ uptake through this transporter (Takahashi et al., 2007a). Another *P. australis* protein, PhaHAK5, could also be involved in Na^+^ transport, as suggested from heterologous expression in yeast (Takahashi et al., 2007b). Recently, a *bona fide* high-affinity Na^+^-selective transporter of higher plants, ZmHAK4, has been identified in maize (Zhang et al., 2019). *ZmHAK4* is predominantly expressed in the root vascular tissue. Knock-out mutants and natural hypomorphic alleles with reduced expression of this gene had increased Na^+^ contents in shoot and xylem sap, and reduced root Na^+^ content under high-Na^+^ conditions. Thus, ZmHAK4 appears to promote shoot Na^+^ exclusion and salt tolerance by retrieving Na^+^ from xylem sap and preventing root-to-shoot Na^+^ translocation (Figure 4). *HAK4* orthologs in rice and wheat are also preferentially expressed in the root stele and encode Na^+^-selective transporters. Thus, *HAK4* orthologs in cereals probably constitute a conserved salt-tolerance mechanism governing Na^+^ delivery to shoots. Last, HvHAK1 of barley (*Hordeum vulgare*) and the rice OsHAK2 could also be involved in Na^+^ influx (Santa-Maria et al., 1997; Horie et al., 2011).

In parallel with HAK transporters, several HKTs also contribute to Na^+^ uptake from the soil, functioning as Na^+^:K^+^ symporters or as Na^+^ uniporters at high Na^+^ concentrations (reviewed by Benito et al., 2014; Hamamoto et al., 2015; Figure 4). Plant HKT proteins display a core structure similar to that of the K^+^ transporter TrkH from *Vibrio parahaemolyticus* (Cao et al., 2011), comprising eight transmembrane (TM) and four pore-forming (P) domains successively arranged in four TM1-P-TM2 motifs in a single polypeptide chain. The assembly of these four TM1-P-TM2 motifs results in the formation of a central permeation pathway similar to that of tetrameric *Shaker-*like K^+^ channels. The plant HKT family has been divided into at least two classes based on a distinguishing feature that lies in the selectivity filter. Class-I (HKT1) members are ubiquitous in plants, mostly Na^+^-selective, and often involved in Na^+^ recirculation through vascular tissues (Maathuis, 2014; Very et al., 2014; Figure 4). Most members of this clade have a highly conserved serine (SGGG motif) in the first pore-loop domain of the protein, while class-II (HKT2) members, found exclusively in monocots, have a glycine instead of the serine (GGGG motif) in this domain and are generally permeable to both Na^+^ and K^+^ (Hauser and Horie, 2010). This classification is not strict as HKT proteins may display different permeation modes depending on the external concentrations of Na^+^ and K^+^. At concentrations of Na^+^ and K^+^ below 1–10 mM, these transporters function essentially as Na^+^:K^+^ symporters (Rubio et al., 1995; Haro et al., 2005), whereas at high external Na^+^ concentrations, above 1–10 mM, HKTs lose their permeability to K^+^ and become Na^+^ uniporters (Gassman et al., 1996; Horie et al., 2001; Jabnoune et al., 2009). On the other hand, an increase in K^+^ concentration reduces the transport rate of both the Na^+^:K^+^ symport and Na^+^ uniport modes (Gassman et al., 1996; Garciadeblas et al., 2003; Jabnoune et al., 2009). These different permeation mechanisms have been explained through two mechanistic models: (i) carrier-mediated transport by an alternating-access model (Gassman et al., 1996; Rubio et al., 1999; Haro and Rodriguez-Navarro, 2002) and (ii) a pore-mediated model very similar to that of K^+^ channels (Durell and Guy, 1999; Tholema et al., 2005; Corratge et al., 2007). The first mechanism posits the existence of two high-affinity binding sites, named K^+^- and Na^+^-coupling sites. In this model, both binding sites need to be occupied for uptake to occur (Rubio et al., 1995; Jabnoune et al., 2009). Thus, the competitive binding of K^+^ and Na^+^ at the K^+^-coupling site would explain both permeation modes, Na^+^:K^+^ symport or Na^+^ uniport. On the other hand, high external K^+^ could inhibit the symport activity assuming that the binding of K^+^ at the Na^+^ coupling site results in a non- or weakly conductive state (Jabnoune et al., 2009). While this mechanism can readily explain the different uniport and symport modes, it does not explain the large currents measured for different HKTs in oocytes for Na^+^ and/or K^+^ (5–10 μA) (Oomen et al., 2012). Considering that the turnover rate reaches values around 10^6^ ions per second, it is likely that HKTs have a pore and they function as ion channels. According to the channel-like model of transport, Na^+^ ions would be bound by two coordination sites in a partially dehydrated form, i.e., retaining only its first hydration shell (Benito et al., 2014). One or two water molecules of the shell might be substituted with polar oxygens of the side chain of the serine residue in the SGGG signature in the first P-loop region. The flexible hydration shell of K^+^ would also allow the coordination of this ion in the two coordination sites. Thus, the molecular permeation model proposed for HKTs, based on Na^+^ permeation in a channel-like structure, could account for the different transport modes observed in the HKT, namely Na^+^ uniport, Na^+^:K^+^ symport and K^+^ uniport (Benito et al., 2014).

An interesting example of the fuzzy classification of HKT proteins is that of two class-II HKT proteins of rice, OsHKT2;1, isolated from Nipponbare, and OsHKT2;2 present in the salt-tolerant Pokkali cultivar. Both proteins share high homology (91%), and yet they exhibit differential Na^+^:K^+^ transport selectivity when expressed in heterologous expression systems. OsHKT2;1 mediates mainly Na^+^ uptake (Jabnoune et al., 2009; Yao et al., 2010), whereas OsHKT2;2 transports both K^+^ and Na^+^ (Horie et al., 2001, 2007). Protein OsHKT2;2 has the typical four Gly residues (GGGG motif) of class-II HKT transporters, permeates both K^+^ and Na^+^ in a large range of concentrations, and functions preferentially as a Na^+^:K^+^ symport, and with low concentration of K^+^ ions exerting an stimulating effect on Na^+^ transport (Horie et al., 2001; Yao et al., 2010; Oomen et al., 2012; Riedelsberger et al., 2018). However, OsHKT2;1 is an atypical HKT class-II member because it contains the SGGG signature in the first pore-loop and mediates selective Na^+^ uptake, which are typical features of class-I HKT transporters (Horie et al., 2001; Garciadeblas et al., 2003). OsHKT2;1 enables Na^+^ uptake into K^+^-starved roots, thereby compensating for the lack of K^+^ as cellular osmolyte (Horie et al., 2007). In wheat, *TaHKT2;1* is preferentially expressed in the root cortex and induced by K^+^ deficiency (Schachtman and Schroeder, 1994) and seems to have a function similar to that of OsHKT2;1 (Horie et al., 2009). *HvHKT2;1* from barley (*Hordeum vulgare*) is also induced by K^+^ deficiency, and the protein demonstrated the co-transports Na^+^ and K^+^ over a large range of concentrations (Mian et al., 2011; Hmidi et al., 2019). Together, these result suggest that HKT2;1 proteins may contribute both to the K^+^ uptake in the presence of Na^+^, and to Na^+^ uptake for osmotic adjustment.

Of note is that substrate selectivity of HKT1 transporters could be modified by single amino acid changes outside the SGGG/GGGD motif dichotomy. Using 3D comparative modeling, Cotsaftis et al. (2012) suggested that K^+^ can be transported unfavorably in class-I members due to a steric hindrance imposed through the G to S substitution, while the G in class-II HKTs would facilitate the transport of K^+^, although under certain conditions these proteins also could transport Na^+^ (Maser et al., 2002a). Some exceptions to this general rule are EcHKT1;2 from *Eucalyptus camaldulensis*, EsHKT1;2 from *Eutrema salsugineum* (formerly *Thellungiella salsuginea or T. halophila)*, SpHKT1;2 from *Schrenkiella parvula* (formerly *T. parvula)*, and McHKT1;1 from *Mesembryantemum crystallinum*, all of which have a Ser in the first pore-loop domain and are permeable to K^+^ (Fairbairn et al., 2000; Su et al., 2003; Jabnoune et al., 2009; Ali et al., 2012). This indicates that K^+^ permeability in HKTs does not depend only on the Gly residue at the pore. Indeed, the alignment of HKTs homologs from *Arabidopsis*, *Eutrema* and *Schrenkiella* species with ScTRK1, a high-affinity potassium transporter of *Saccharomyces cerevisiae*, showed that both EsHKT1;2 (*E. salsugineum*) and SpHKT1;2 (*S. parvula*) contained, alike ScTRK1, conserved Asp residues in their second pore-loop domains (Asp207 and Asp205, respectively) (Ali et al., 2012). However, in most HKT1-like proteins an Asn is present at the corresponding position. The change of Asp207 to Asn207 in EsHKT1;2 and Asp205 to Asn205 in SpHKT1;2 abolished K^+^ uptake and generated the typical Na^+^-selective transport of class-I HKTs (Ali et al., 2012, 2018). Moreover, changing the Asn residue in the 2nd pore-loop domain of AtHKT1 to Asp, converted a highly selective Na^+^ transporter into a transporter more similar to EsHKT1;2, with high affinity for K^+^. Transgenic *Arabidopsis* plants that expressed the AtHKT1-Asn211Asp variant were more tolerant to salt stress than controls with wild type AtHKT1, and showed the same tolerance phenotype than having EsHKT1;2 or SpHKT1;2 overexpressed in *Arabidopsis* plants (Ali et al., 2016, 2018). Consequently, Ser in the SGGG motif of the first pore-loop domain appears not to be the only essential amino acid favoring Na^+^ uptake (at least in *Arabidopsis*, *Eutrema*, and *Schrenkiella* species), but it possibly functions as a supporting residue. All these examples show that the cation selectivity of HKT transporters could be convertible by exchanging single amino acids, and that structural elements localized in regions outside the selectivity filter can determine the ionic selectivity for Na^+^ and/or K^+^ of HKT proteins.

Notably, mutations inactivating Na^+^-selective HKT1-like transporters reduce the K^+^ contents of shoots during salt exposure. For instance, mutations of *hkt1* in Arabidopsis cause opposite effects on the K^+^ content with respect to that of Na^+^ both in roots and shoots, maintaining lower K^+^ levels in shoots but higher K^+^ in roots (Maser et al., 2002b; Sunarpi et al., 2005). In rice, the *SKC1* locus identified as a QTL for shoot K^+^ content encodes the Na^+^-selective protein HKT1;5 whose activity, however, determines the accumulation of K^+^ in aerial parts (Ren et al., 2005). A similar situation has been described for the salt-tolerance *NAX2* locus of wheat, also encoding an HKT1;5 protein (Munns et al., 2012). These results suggest a connection between Na^+^ unloading via HKT1-like proteins and K^+^ loading from xylem parenchyma cells under salt stress. This phenomenon could be explained if the uptake of Na^+^ through HKT1 proteins caused membrane depolarization in xylem parenchyma cells, thereby promoting the opening of outward-rectifying K^+^ channels, such as SKOR, and the K^+^ accumulation in the xylem and leaves (Horie et al., 2009). SKOR allows of K^+^ release into the xylem vessels from xylem parenchyma cells (Gaymard et al., 1998). Together, these results suggest that HKT1-like proteins provide two essential mechanisms toward mediating salt tolerance: (i) prevention of Na^+^ over-accumulation in leaves; and (ii) allowing the K^+^ accumulation in leaves through outward-rectifying K^+^ channels.

Additional pathways for Na^+^ entry in plant cells may be provided by Non-Selective Cation Channels (NSCC) (Figure 4). Negative electrical membrane potential and high extracellular Na^+^ concentrations promote passive entry of Na^+^ into roots through ion channels. Electrophysiological experiments in *A. thaliana* protoplasts have shown that NSCCs could be Na^+^ influx pathways (Demidchik and Tester, 2002; Tyerman, 2002). These proteins form a heterogeneous group of plasma membrane channels with a high selectivity for cations over anions, while differing in their ability to conduct mono- and divalent cations (Tyerman, 2002; Zhang et al., 2002; Demidchik et al., 2002a, b; Demidchik and Maathuis, 2007). NSCC channels are classified into three major families according to their response to changes in membrane electrical potential: depolarization-activated NSCCs (DA-NSCCs), hyperpolarization-activated NSCCS (HA-NSCCs) and voltage-insensitive NSCCs (VI-NSCCs) (Demidchik and Maathuis, 2007). This last group is commonly found in plasma membrane of roots and leaves of different plant species (Tyerman et al., 1997; Demidchik and Tester, 2002; Demidchik et al., 2002b; Shabala et al., 2006, 2007; Zhao et al., 2007, 2011; Velarde-Buendia et al., 2012). VI-NSCCs weakly differentiate among different cations, with the preference K^+^ > NH^+4^ > Rb^+^ ∼ Cs^+^ ∼ Na^+^ > Li^+^ > tetraethylammonium (TEA^+^). In general, they have significant Na^+^ conductance, but still lower than that of K^+^ (Kronzucker and Britto, 2011). Cyclic nucleotide-gated channel (CNGC), have been suggested to be VI-NSCCs channels (Maathuis and Sanders, 2001; Demidchik et al., 2002b; Demidchik and Maathuis, 2007), or weakly voltage-sensitive (Leng et al., 2002; Lemtiri-Chlieh and Berkowitz, 2004; Wang et al., 2013; Mori et al., 2018). CNGCs permit the diffusion of monovalent and divalent cations such as Na^+^, K^+^, and Ca^2+^ (Leng et al., 1999, 2002; Demidchik and Maathuis, 2007; Mian et al., 2011; Hanin et al., 2016). They are ligand-gated channels regulated by reversible binding of adenosine 3′,5′-cyclic monophosphate (cAMP), guanosine 3,5-cyclic monophosphate (cGMP) (Balague et al., 2003; Chin et al., 2009; Ramanjaneyulu et al., 2010), or calmodulin (CaM) to the cyclic nucleotide binding domain (Kohler and Neuhaus, 2000; Hua et al., 2003). In fact, the first *CNGC* gene in plants was identified during a screen for CaM binding partners in *Hordeum vulgare* (Schuurink et al., 1998). Subsequently, 20 CNGC family members have been identified in *A. thaliana* (Kohler et al., 1999), 16 in rice, *Oryza sativa* (Nawaz et al., 2014), 18 in tomato, *Solanum lycopersicum* (Saand et al., 2015), 21 in pear, *Pyrus bretschneideri* (Chen et al., 2015) and 26 in the Chinese cabbage *Brassica oleracea* (Kakar et al., 2017). The largest family was recently described in wheat, *Triticum aestivum*, with 47 *TaCNGC* genes (Guo et al., 2018). These proteins share structural homology with *Shaker*-like channels, with six transmembrane segments and a long cytosolic C-terminal domain harboring a cyclic nucleotide-binding domain. However, they lack the canonical motif TxGYG, a hallmark of K^+^-selective channels (Talke et al., 2003; Szczerba et al., 2009). All CNGCs of *P. bretschneideri* and *A. thaliana* contain positively charged residues in the S4 motif, similar to voltage-dependent K^+^ channels (Chen et al., 2015). Likewise, in HvCNGC2-3, four arginine residues and a lysine are present through S2 to S4 (Mori et al., 2018). Thus, it is possible that the voltage sensitivity observed in some CNGCs could discredit a significant involvement in mediating Na^+^ fluxes for extended periods of time (Kronzucker and Britto, 2011). More electrophysiological experiments are required to determine the real importance of the charged residues in the voltage sensitivity of these channels. Moreover, salt stress increases cGMP level in *Arabidopsis* roots, thereby inhibiting the permeability of CNGC channels to Na^+^ and reducing its entry to root cells (Maathuis and Sanders, 2001; Donaldson et al., 2004). Together, these findings question that CNGC could represent a significant pathway for Na^+^ entry.

Some members of *A. thaliana*, like AtCNGC2, appears to be selective for K^+^ over other alkali metal cations (Cs^+^, Li^+^, and Rb^+^) and to exclude Na^+^ (Leng et al., 2002), while others are able to transport both K^+^ as well as Na^+^, thereby impacting on cytosolic K^+^:Na^+^ ratios under saline conditions. AtCNGC3 is mostly expressed in epidermal and cortical root tissues. The loss of function of CNGC3 alters the ionic composition of seedlings of *Arabidopsis*, reducing the net Na^+^ uptake and promoting K^+^ accumulation (Gobert et al., 2006). AtCNGC10 is also permeable to Na^+^ and K^+^, and antisense lines exhibited alterations in the content of both cations within roots and shoots (Guo et al., 2008) while overexpression could partially compensate the knockout mutation *akt1-1* inactivating a *Shaker*-type channel implicated in uptake of K^+^ by roots (Li et al., 2005). Recently, electrophysiological analysis of the barley HvCNGC2-3 (*Hordeum vulgare)* has shown that this channel is activated only by the co-presence of K^+^ and Na^+^ (Mori et al., 2018). This property has not been reported for any other CNGC, and although its meaning is still unclear, the root-expressed HvCNGC2-3 could be involved in the response to salinity stress, improving the osmotic adjustment of roots. In the case of barley, the permeability of Na^+^ and K^+^ by CNGC2-3 could have a role in balancing the ratio of these cations in the cells sustaining osmotic potential in the roots.

As mentioned before, Na^+^ can partially substitute for K^+^ as a cellular osmolyte, particularly in conditions in which K^+^ is limiting. The Na^+^ acquired for osmotic purposes must be sequestered within the vacuoles to avert its cytotoxic effect in the cytosol and other intracellular components (Mittler and Blumwald, 2010). Cation/H^+^ antiporters are thought to mediate the transport of Na^+^ into the vacuole, driven by the electrochemical gradient of protons generated by the vacuolar ATPase (V-ATPase) and pyrophosphatase (V-PPase) enzymes (Sze and Chanroj, 2018; Shabala et al., 2019; Figure 4). Na^+^/H^+^ exchange is mediated by members of a family of transporters referred to as Na^+^-H^+^ exchangers, named NHXs in plants and NHEs in animals (Jiang et al., 2010; Chanroj et al., 2012). However, detailed biochemical and molecular genetic analyses have shown that members of the plant NHX family have different ion selectivities that correlate with the cellular membrane in which they are placed (Jiang et al., 2010; Chanroj et al., 2012; Ragel et al., 2019). Thus, the plasma membrane localized proteins SOS1/NHX7 and NHX8 show great selectivity for Na^+^ and Li^+^, respectively, and they are involved in the plant tolerance to high levels of these cations (Shi et al., 2002; An et al., 2007; Quintero et al., 2011), whereas family members sorted to endosomal membranes show various degrees of non-selective transport of the monovalent alkali cations Na^+^, K^+^ and Li^+^ (Jiang et al., 2010; Huertas et al., 2013; Bassil et al., 2019; Figure 4).

Shortly after the identification of plant NHXs, Apse et al. (1999) showed that overexpression of the vacuolar isoform *AtNHX1* of *Arabidopsis* increased salinity tolerance and greater Na^+^/H^+^ exchange activity in isolated leaf vacuoles. Although this first report concluded that AtNHX1 was specific to Na^+^ transport, later studies have shown that AtNHX1 and other tonoplast localized NHXs mediate vacuolar K^+^ uptake under normal growth conditions and in the presence of moderate Na^+^ concentrations (Venema et al., 2002; Leidi et al., 2010; Bassil et al., 2011; Barragan et al., 2012; Andres et al., 2014). Overexpression of *AtNHX1* in tomato plants promoted higher vacuolar K^+^ content under different growth conditions, and increased the salinity tolerance of transgenic plants via retention of intracellular K^+^ and without influencing vacuolar Na^+^ accumulation (Leidi et al., 2010). Similarly, transgenic alfalfa overexpressing the wheat TaNHX2 exchanger, a vacuolar isoform, decreased K^+^ efflux by reducing plasma membrane depolarization and activation of K^+^ outwardly rectifying channels, thereby retaining more intracellular K^+^ under salt stress conditions (Zhang et al., 2015). LeNHX2 protein, which is preferentially localized in non- endomembranes from tomato, catalyzes specifically K^+^/H^+^ antiport in proteoliposomes, showing very low activities with other monovalent cations, including Na^+^ (Venema et al., 2003; Huertas et al., 2013). Endosomes have been identified as a target for Na^+^ toxicity (Hernandez et al., 2009) and it has been suggested that intracellular non-vacuolar isoforms, such as LeNHX2 or Arabidopsis NHX5 and NHX6 could have greater selectivity for K^+^ over Na^+^ as to prevent excessive Na^+^ uptake into endosomes (Hernandez et al., 2009; Jiang et al., 2010), whereas those NHXs localized to the tonoplast would not discriminate since their main function is to accumulate ions in the vacuolar lumen for osmotic adjustment, cell turgor and control of the vacuolar pH (Venema et al., 2002; Bassil et al., 2011; Barragan et al., 2012; Andres et al., 2014).

Recently, multiple knockout mutants of Arabidopsis lacking all but one of the four vacuolar isoforms (NHX1, NHX2, NHX3, NHX4) and quadruple knockout plants lacking all vacuolar NHX activity, have been analyzed (Bassil et al., 2019). Kinetic analysis of K^+^ and Na^+^ transport indicated that NHX1, NHX2, and NHX4, are the main transporters of K^+^ in the vacuoles, while AtNHX3 could mediate Na^+^ transport. The lack of NHX activity at the tonoplast (*nhx1*–*nhx4*) resulted in no K^+^ uptake and in highly acidic vacuolar lumen. This mutant displayed Na^+^ transport with an apparent Km of 9.9 mM, suggesting the existence of an alternative, cation/H^+^-independent mechanism that permitted the transport of Na^+^ into vacuoles, as previously suggested (Barragan et al., 2012). These results confirm a large amount of evidence demonstrating the polyvalent role of NHX as Na^+^/H^+^ and/or K^+^/H^+^ exchangers in vacuolar membranes. Refined structural modeling combined with the identification of amino acid residues involved in ion coordination and transport (Wang et al., 2015) could allow the rational design of Na^+^-selective tonoplast-localized NHXs that would be instrumental in achieving salt tolerance based on efficacious Na^+^ sequestration into vacuoles. This strategy should most likely be combined with the reduction of Na^+^ leaks back to the cytosol through vacuolar channels (Shabala et al., 2019).

## Author Contributions

All authors have contributed to literature search, discussion, and writing of the manuscript. LM prepared the figures. JP assembled all the sections. All authors checked and approved the manuscript.

## Conflict of Interest

The authors declare that the research was conducted in the absence of any commercial or financial relationships that could be construed as a potential conflict of interest.
